# Supplementary material for: Biochemical Characterization and In Vitro Digestibility of Protein Isolates from Hemp (Cannabis sativa L.) By-Products for Salmonid Feed Applications
Source: Molecules. 2022 Jul 27;27(15):4794. doi: 10.3390/molecules27154794 (PMC9369619; doi:10.3390/molecules27154794)
Supplement: Supplementary file 1 [file molecules-27-04794-s001.zip › molecules-1827531-supplementary.pdf]

SUPPLEMENTRY DOCUMENT

Biochemical Characterization and *in vitro* Digestibility of Protein Isolates from Hemp (*Cannabis sativa* L.) By-Products for Salmonid Feed Applications

(A)

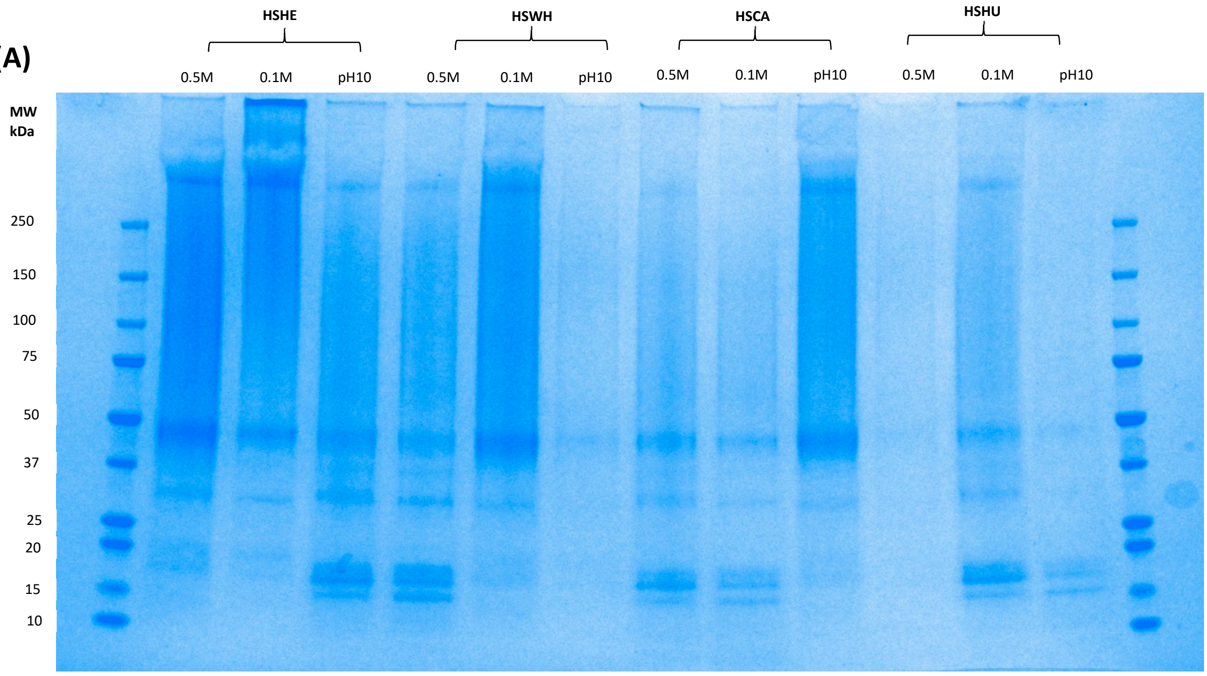

(B)

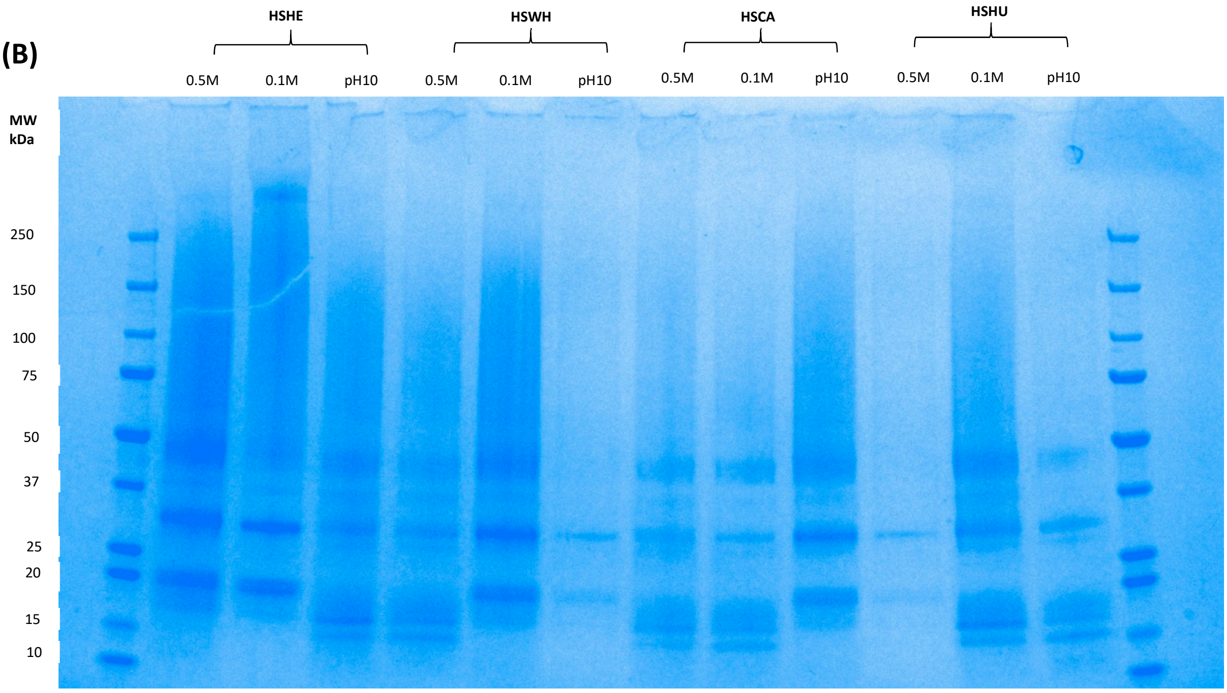

Figure S1. Non-reducing (A) and reducing (B) SDS-PAGE profiles for Protein isolates extracted hemp seeds and hemp by-products. HSHE - hemp hearts; HSWH – hemp whole seed; HSCA – hemp cake and HSHU – hemp seed hulls

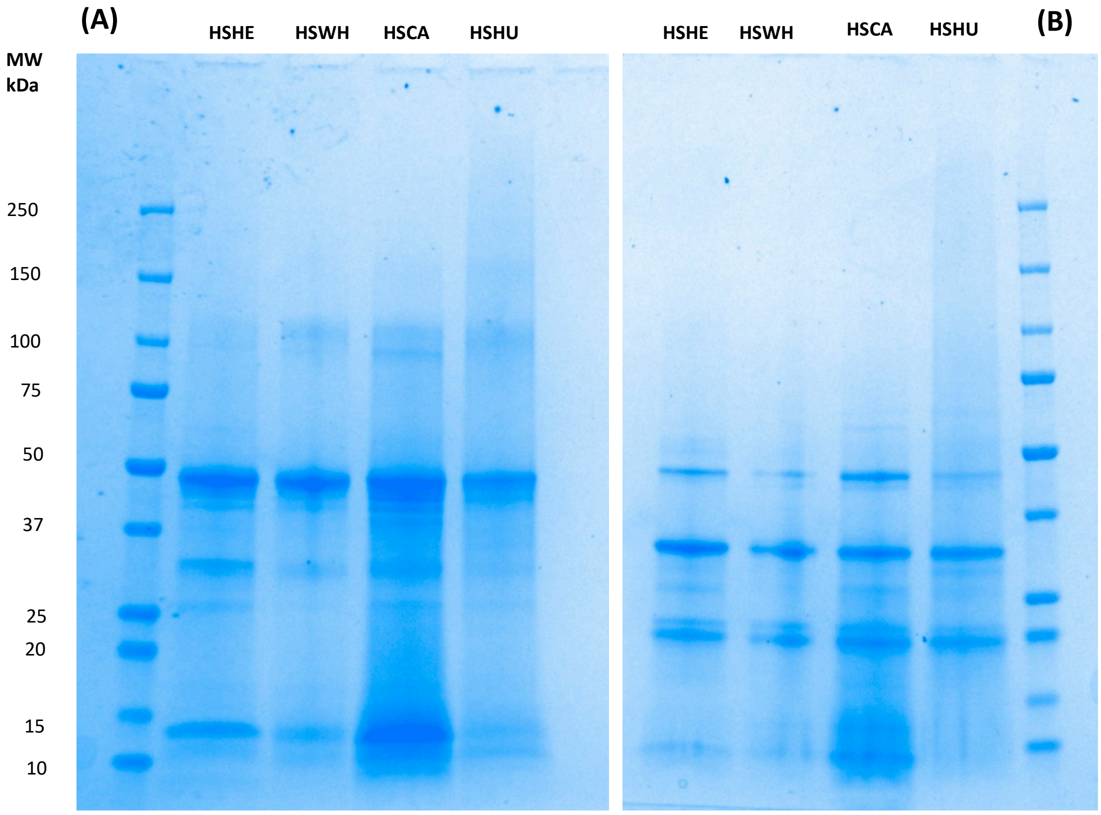

Figure S2. Non-reducing (A) and reducing (B) SDS-PAGE profiles for Protein isolates extracted hemp seeds and hemp by-products extracted at pH 10.0. HSHE - hemp hearts; HSWH – hemp whole seed; HSCA – hemp cake and HSHU – hemp seed hulls

(A)

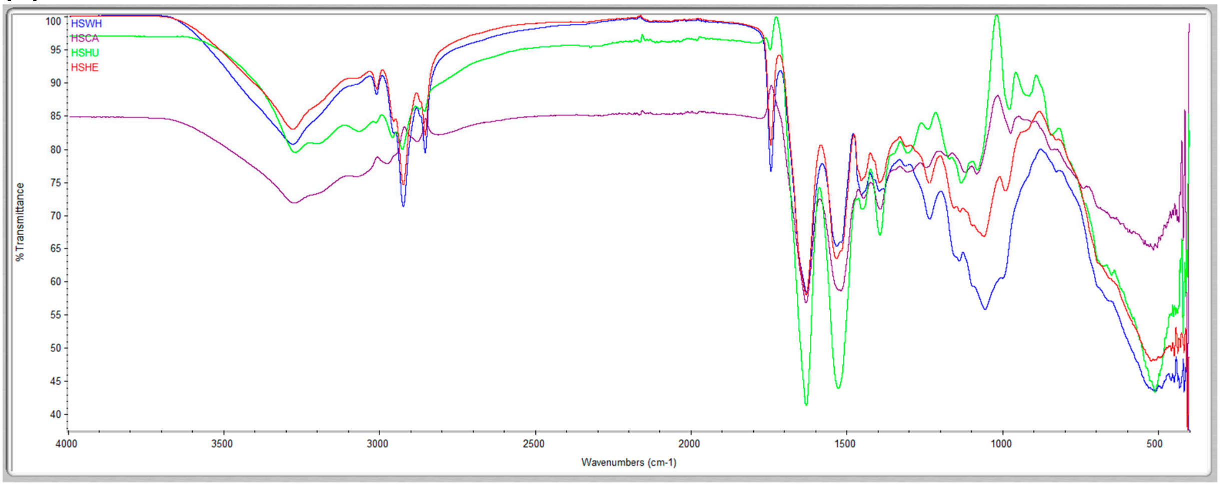

(B)

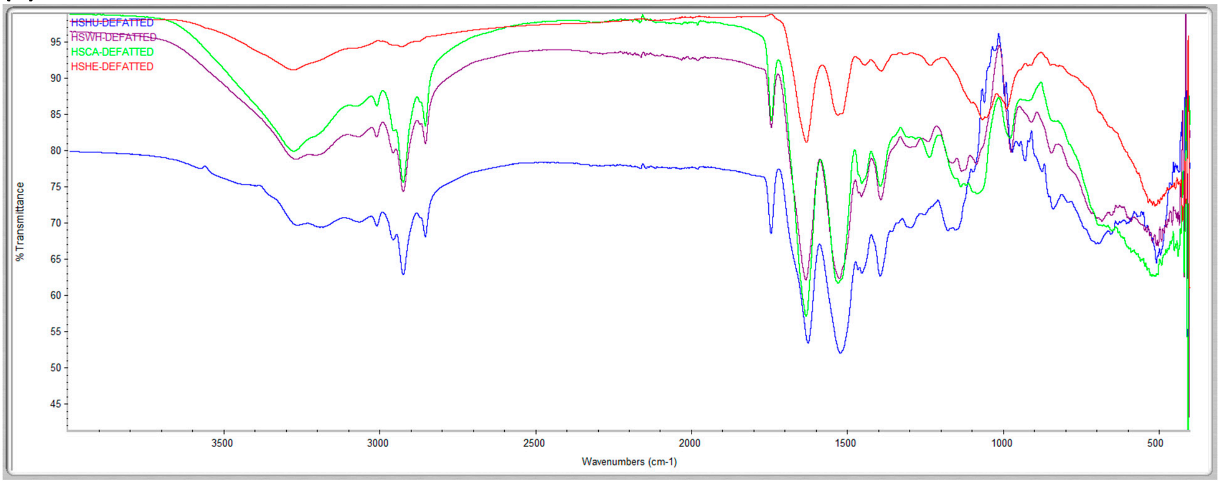

(C)

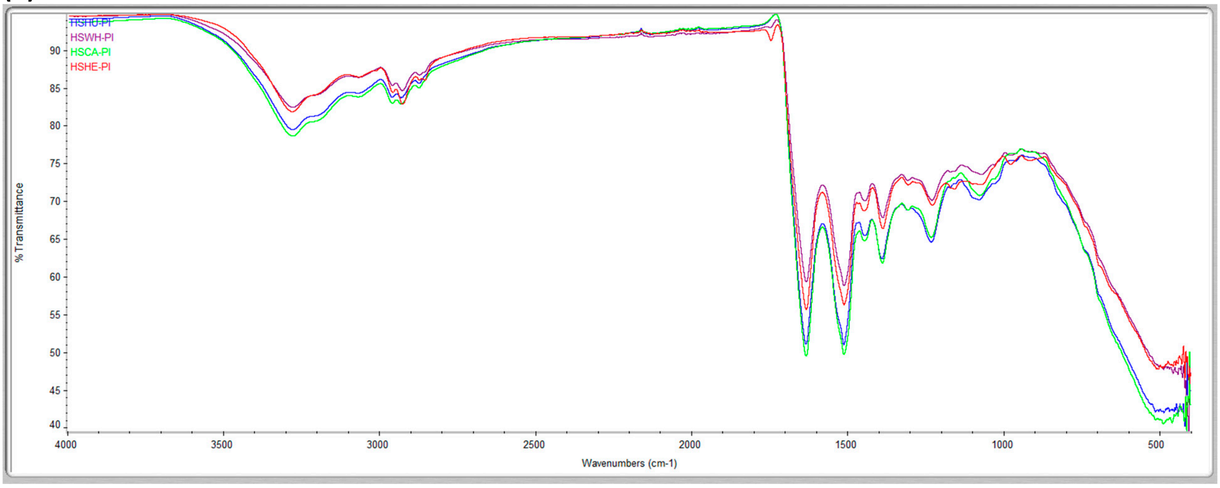

Figure S3. IR spectrum of hemp cake, hemp hulls, hemp whole seeds and hemp hearts. (A) Original biomass (B) Defatted biomass and (C) protein isolate (PI). HSHE - hemp hearts; HSWH – hemp whole seed; HSCA – hemp cake and HSHU – hemp seed hulls.

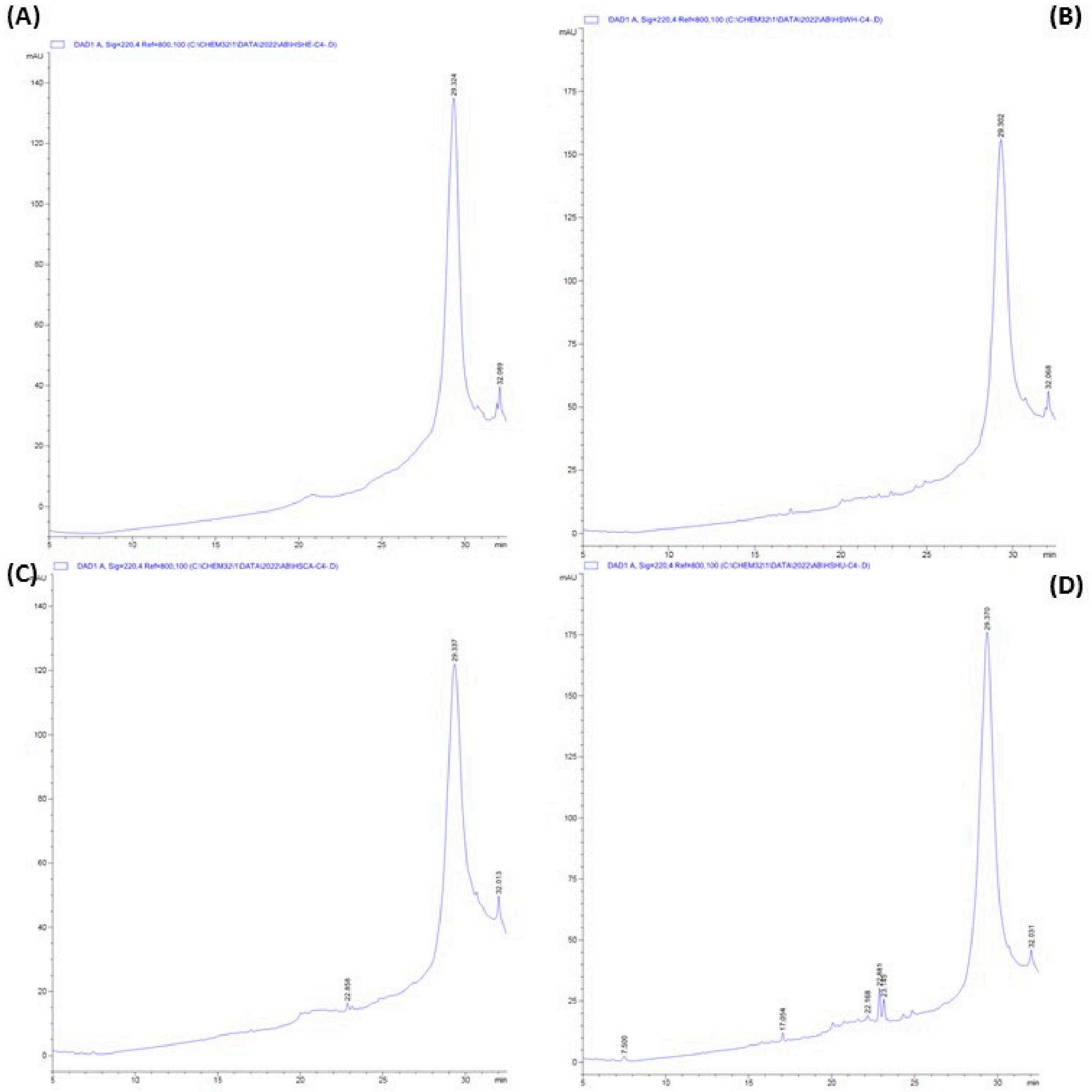

Figure S4. HPLC chromatogram of protein isolates extracted from (A) hemp hearts (B) hemp whole seed (C) hemp cake and (D) hemp seed hulls. HPLC was carried out on an Agilent 1100 Series HPLC equipped with a diode array detector. HPLC condition column - ACE C4-300 (4.6µm, 150 mm ACE Aberdeen, UK); eluent - 0.1% TFA in CAN:0.1 TFA in Milli-Q water 5:95 (v/v) 0-5 min; 5:95-35:65 (v/v) 5-25 min, 35:65-50:50 (v/v) 25-30 min and back to initial equilibrium at 35 min, flow rate – 1.0 mL/min and detection UV at 220 nm.

Table S1. Amino acid analysis of hemp protein isolates extracted with 0.5M NaOH and at pH 10.0 (results expressed in mg/g)

Amino acid/	HSHE		HSWH		HSCA		HSHU	
	0.5 M	pH 10.0**	0.5 M	pH 10.0**	0.5 M	pH 10.0	0.5 M	pH 10.0
Histidine (His)*	32.7 ± 0.6	25.5 ± 0.7	27.7 ± 1.5	26.5 ± 2.1	21.7 ± 0.6	25.7 ± 1.5	20.0 ± 0.0	23.7 ± 1.2
Serine (Ser)	11.0 ± 2.0	14.0 ± 0.0	12.0 ± 2.6	11.5 ± 0.7	20.7 ± 6.4	9.7 ± 1.2	8.3 ±0.6	10.7 ± 0.6
Arginine (Arg)*	107.7 ± 0.6	124.5 ± 6.4	96.0 ± 4.6	135.0 ± 5.7	96.7 ±2.5	116.0 ± 7.8	86.3 ± 2.1	110.3 ± 6.1
Glycine (Gly)	47.0 ± 0.0	40.5 ± 2.1	41.7 ± 1.5	41.0 ± 1.4	34.0 ± 1.0	37.0 ± 2.6	34.3 ± 1.2	38.3 ± 2.5
Asparate (Asp)	105.7 ± 1.5	94.0 ± 7.1	87.3 ± 3.3	102.5 ± 3.5	94.7 ± 7.2	83.3 ± 8.1	62.3 ± 3.1	83.3 ± 4.5
Glutamate (Glu)	130.3 ± 2.1	157.0 ±	120.3 ± 3.1	174.0 ± 4.2	144.0 ± 8.7	154.3 ± 14.2	120.7 ± 5.1	142.0 ± 7.5
Threonine (Thr)*	16.0 ± 1.0	19.5 ± 0.7	15.7 ± 1.5	17.0 ± 1.4	20.3 ± 3.2	14.0 ± 1.0	13.0 ± 0.0	16.3 ± 0.6
Alanine (Ala)	48.7 ± 0.6	38.0 ± 2.8	42.0 ± 1.0	38.0 ± 1.4	38.3 ± 2.3	30.3 ± 2.9	25.3 ± 1.2	34.3 ± 1.5
Proline (Pro)	39.3 ± 0.6	34.0 ± 1.4	35.3 ± 0.6	34.5 ± 0.7	31.0 ± 1.0	30.7 ± 2.3	24.7 ± 0.6	32.0 ± 2.0
Cysteine (Cys)	-	3.0 ± 1.4	-	3.0 ± 1.4	-	3.0 ± 0.0	-	2.0 ± 0.0
Lysine (Lys)*	26.3 ± 1.2	28.5 ± 2.1	26.7 ± 2.9	25.5 ± 2.1	31.3 ± 3.2	24.0 ± 4.0	14.7 ± 0.6	25.3 ± 1.5
Tyrosine (Tyr)	37.3 ± 0.6	26.5 ± 0.7	31.3 ± 3.1	26.0 ± 2.8	24.0 ±2.6	22.0 ± 0.0	21.0 ± 0.0	22.3 ± 1.2
Methionine (Met)*	32.0 ± 0.0	20.0 ± 1.4	30.0 ± 1.0	20.5 ± 0.7	19.3 ±0.6	22.3 ± 1.2	19.0 ± 1.7	19.0 ± 1.0
Valine (Val)*	74.7 ± 0.6	53.5 ± 3.5	62.7 ± 1.2	54.5 ± 0.7	50.7 ±2.1	44.0 ± 3.5	33.7 ± 1.5	49.3 ± 2.5
Isoleucine (Ile)*	51.7 ± 0.6	45.0 ± 2.8	45.7 ± 1.2	45.0 ± 0.0	40.3 ± 1.5	37.3 ± 2.9	28.7 ± 1.5	40.7 ± 2.1
Leucine (Leu)*	83.3 ± 1.2	64.0 ± 4.2	72.7 ± 1.2	63.5 ±0.7	60.3 ± 2.5	53.7 ± 4.0	42.3 ± 2.1	59.3 ± 3.5
Phenylalanine (Phe)*	60.3 ± 5.3	47.5 ± 2.1	52.0 ± 3.6	48.0 ± 2.8	38.3 ± 0.6	40.3 ± 2.1	30.7 ± 0.6	43.0 ± 2.6
**Total (mg/g)**	**904.0 ± 5.3**	**835.0 ±**	**799.0 ± 17.6**	**866.0 ± 9.9**	**765.7 ± 43.7**	**747.7 ± 57.7**	**585.0 ± 20.0**	**752.0 ± 40.0**
**Crude Protein (% N x 5.37)**	**85.7 ± 0.2**	**83.9 ± 0.1**	**79.2 ± 0.3**	**91.2 ± 0.1**	**75.9 ± 0.2**	**87.3 ± 0.2**	**69.7 ± 0.3**	**83.3 ± 0.1**

* Essential amino acids for fish, ** data from duplicate experiment, (-) not detected, HSHE - hemp hearts; HSWH – hemp whole seed; HSCA – hemp cake and HSHU – hemp seed hulls.
